# Evolutionary medicine – the quest for a better understanding of health, disease and prevention

**DOI:** 10.1186/1741-7015-11-116

**Published:** 2013-04-29

**Authors:** Martin Brüne, Ze’ev Hochberg

**Affiliations:** 1 Division of Cognitive Neuropsychiatry and Psychiatric Preventive Medicine, LWL University Hospital, Ruhr-University Bochum, Alexandrinenstrasse 1, Bochum, D-44791, Germany; 2 Rappaport Family Faculty of Medicine and Research Institute, Meyer Children’s Hospital, Rambam Med Center, Technion-Israel Institute of Technology, Haifa, 30196, Israel

## Abstract

Clinical medicine has neglected the fact that the make-up of organs and body functions, as well as the human-specific repertoire of behaviors and defenses against pathogens or other potential dangers are the product of adaptation by natural and sexual selection. Even more, for many clinicians it does not seem straightforward to accept a role of evolution in the understanding of disease, let alone, treatment and prevention.

Accordingly, this Editorial seeks to set the stage for an article collection that aims at dealing precisely with the question of why evolutionary aspects of health and disease are not only interesting, but necessary to improve clinical medicine.

## Editorial

Clinicians take for granted that any body part can go wrong at some point in life, as much as a faulty gadget that is poorly adapted to the burden of life [1]. Why is this so? From an evolutionary perspective, it is all but straightforward to assume that the human body is fraught with thousands of flaws that make us vulnerable to disease. Selection has shaped the functional properties of organs over eons close to optimum to convey fitness advantages in terms of survival and reproduction. If evolution by natural selection has sculpted our exquisite design, why do our lungs, hearts, brains and other parts of the body become so often disarrayed? For example, if organs operate well for 50 years, why cannot they continue to do so for another 50 years? Why do our built-in defense mechanisms break down from time to time? After all, why do we get sick at all, as Nesse and Williams inquired some 20 years ago [2]?

Evolutionary medicine offers important insight to these and similar questions; in fact, we assert that questions concerning the causes of sickness and disease cannot be answered without an evolutionary approach. Nor can medicine promote preventive action to help people maintain health without a profound knowledge of gene-environment interactions that were shaped in our distant past.

A common medical misconception is to assume that any organ or body part is meant to be perfect by design, ultimately to ascertain longevity, whereas the view offered by Darwinian evolution is that organisms are ‘designed’ to secure the propagation of genes coded in the DNA. Design compromises, produced by opposing selection pressures on the same structure, may cause apparent suboptimal design. The most famous example in the evolutionary literature is probably that of the peacock’s tail, which makes peacocks sexually attractive to peahens, but also puts the peacock at risk of being devoured by predators [3], an evolutionary process referred to as the ‘handicap principle’ [4]. Another example is bipedalism, which putatively evolved to enable our hominin ancestors to travel long distances at relatively low energy consumption [5], but renders humans vulnerable to develop instability of the vertebral column (slipping discs). Walking upright also produced a discrepancy between the growing newborn skull and the narrowing maternal birth canal, which forced our ancestors to deliver birth to immature and helpless babies (“the obstetrical dilemma”, [6]) – a design compromise that radically changed the way we care for our infants, with far-reaching consequences for our human psyche, including attachment, trust and cooperation [7]. Thus, the human body (like all other living organisms) comprises a set of design trade-offs of often conflicting adaptive mechanisms [2].

Evolutionary medicine emphasizes the mismatch of humans’ slowly evolving bodies with rapidly changing modern environments [8], providing access to new toxins, such as tobacco, alcohol and other psychotropic substances, as well as to high-calorie, high-fat diets along with an increasingly sedentary and physically less active lifestyle [9]. In addition, environmental pollution, excessive population growth producing social stress, the introduction of effective methods of birth control, and the changing patterns of exposure to infectious agents [10] have all left their marks on our bodies and continue to do so. The catalogue of conditions that may arise from such mismatch is long, including obesity, metabolic syndrome and Type 2 diabetes, coronary heart disease, Crohn’s disease, renal failure, osteoporosis, stroke, depression, Alzheimer’s disease, atherosclerosis, asthma, cancer, chronic liver disease or cirrhosis, chronic obstructive pulmonary disease, and sexually transmitted infections, to mention just those ranking high on the WHO ‘hit-list’ of diseases causing disability.

One of the most important lessons from evolution for an aging society is to learn why we must age and why so many get cancer. Cancer and aging are not adaptive, so why do they exist? A trade-off conveyed by antagonistic pleiotropy is one of the key concepts that answers this question. It concerns the action of genes that have beneficial effects at early stages of development (particularly when one’s reproductive potential peaks), whereas the same genes exert deleterious effects as the organism ages [11,12].

Now that evolutionary medicine has been around for almost 20 years, we strongly believe the time is ripe for the field to engage in translational research. This encompasses research into evolutionary developmental biology (“evo-devo”), which studies the developmental mechanisms that control body growth, shape and form [13], the alterations in gene expression and function that lead to such phylogeny [14], and issues pertaining to the role of gene-environment interaction in health and disease [15].

In the forthcoming series to be published in *BMC Medicine*, distinguished scholars from various medical backgrounds will demonstrate how insights from evolutionary theory not only improve our understanding of disease, but offer new ways to diagnose, manage and prevent human ailment.

As a par excellence illustration for trade-offs of early-life advantage against later disease, Al-Daghri *et al.* report on positive selection of the *NPC1* gene that has been associated with type-2 diabetes, yet also conveys some protection against virus infection [16]. Brüne has published on genetic polymorphisms of the oxytocin receptor as possible candidates for conveying “differential susceptibility” to developing psychiatric disorders [17]. Hochberg and Belsky examine adolescence as an evolutionary life-history stage in its developmental context. They show that the transition from the preceding stage of juvenility entails adaptive plasticity in response to energy resources, other environmental cues, social needs of adolescence, and maturation toward youth and adulthood. Using the evolutionary theory of socialization, they demonstrate that familial psychosocial stress fosters a fast life-history and reproductive strategy rather than early maturation being just a risk factor for aggression and delinquency [18]. In a similar vein, yet from a translational research perspective, Crispel *et al.* describe in a rat model how the age at weaning from lactation regulates life history, growth, body composition and maturational tempo [19]. Rühli and Henneberg highlight the fact that humans continue to evolve and that microevolutionary changes can be observed over a few generations, including changes that are brought about by improved medical care [20].

This new article collection on Evolutionary Medicine is intended to encourage clinicians to acknowledge the fruitfulness of adding an evolutionary dimension to the “standard” approach of understanding the causes of disease, to advance treatment of disease, and to bolster the search for improved prevention [15,21].

## Competing interests

The authors declare that they have no competing interests.

## Authors’ information

MB is Professor of Cognitive Neuropsychiatry and Psychiatric Preventive Medicine and the author of the *Textbook of Evolutionary Psychiatry. The Origins of Psychopathology*, Oxford University Press, 2008.

ZH is Professor of Pediatrics and Endocrinology and the author of *Evo-Devo of Child Growth*, Wiley, 2012.

## Pre-publication history

The pre-publication history for this paper can be accessed here:

http://www.biomedcentral.com/1741-7015/11/116/prepub
